# Chorioamnionitis and Neonatal Morbidity and Mortality in Extremely Preterm Infants Born at 23–28 Weeks: A Single-Centre Retrospective Study

**DOI:** 10.3390/jcm15124406

**Published:** 2026-06-06

**Authors:** Gabriela C. Zaharie, Monica G. Hăşmăşanu, Ernestine Haralambous, Flaviu A. Zaharie, Anna D. Jakab, Melinda Matyas

**Affiliations:** 1 Neonatology Department, “Iuliu Haţieganu” University of Medicine and Pharmacy, 400006 Cluj-Napoca, Romania; gzaharie@umfcluj.ro (G.C.Z.); melinda.matyas@umfcluj.ro (M.M.); 2 Pediatrics Department, Centre Hospitalier du Valais Romand, 1951 Sion, Switzerland; ernestine.haralambous@gmail.com; 3 Department of General Surgery, Vienna General Hospital, Medical University of Vienna, 1090 Vienna, Austria; zandrei75@yahoo.com; 4 Faculty of Medicine, “Iuliu Hațieganu” University of Medicine and Pharmacy, 400006 Cluj-Napoca, Romania; jakab.anna.dorottya@elearn.umfcluj.ro

**Keywords:** chorioamnionitis, histological chorioamnionitis, extreme prematurity, neonatal morbidity, respiratory distress syndrome, neonatal mortality

## Abstract

**Background/Objectives:** Chorioamnionitis (CA), an inflammation, with or without infection, involving the amniotic fluid, placenta, fetal membranes or decidua, can significantly impact fetal and neonatal development. This study aimed to determine the prevalence of chorioamnionitis and confirm its correlation with neonatal morbidity and mortality, in a single tertiary center. **Methods:** This observational, retrospective study was conducted over three years (2019–2021) in a tertiary neonatal intensive care unit, examining 80 preterm infants born at 23–28 weeks of gestation. Spearman rank correlation, χ^2^ tests, and multivariate logistic regression were used to assess associations between chorioamnionitis exposure and neonatal outcomes. **Results:** Among the 80 newborns analysed, clinical chorioamnionitis was identified in 12 preterm infants, while 65 (81.3%) presented histological chorioamnionitis. No significant association was found between histological chorioamnionitis stage and gestational age at birth (Spearman ρ = −0.15, *p* = 0.195). Premature rupture of membranes was significantly more frequent in the CA-exposed group (46.2% vs. 13.3%, *p* = 0.019). In unadjusted analyses, histological chorioamnionitis exposure was associated with higher rates of adverse neonatal outcomes, including early-onset sepsis (46.2% vs. 26.7%), intraventricular haemorrhage (73.8% vs. 60.0%), bronchopulmonary dysplasia (15.9% vs. 6.7%), and retinopathy of prematurity (11.3% vs. 0.0%); however, most of these differences did not reach statistical significance. After multivariate adjustment, histological chorioamnionitis remained independently associated with severe respiratory distress syndrome (adjusted OR 25.84, 95% CI 2.49–268.44, *p* = 0.006). Mortality was numerically lower in the CA-exposed group (27.7% vs. 46.7%); however, this difference did not reach statistical significance (*p* = 0.216). **Conclusions:** Histological chorioamnionitis was independently associated with severe respiratory distress syndrome. Associations with early onset sepsis, bronchopulmonary dysplasia, and retinopathy of prematurity were observed in unadjusted analyses but were not independently significant after adjustment for perinatal confounders. No significant association was found between chorioamnionitis and neonatal mortality. While clinical diagnostic criteria for chorioamnionitis demonstrated good specificity, their poor sensitivity underscores the urgent need for improved diagnostic tools, including routine histological examination of the placenta.

## 1. Introduction

Chorioamnionitis is defined as an inflammation, with or without infection, of any combination of the amniotic fluid, placenta, fetal membranes or decidua [1]. The term “Triple I” (referring to intrauterine inflammation, or infection, or both) has been proposed to replace the traditional term “chorioamnionitis”, distinguishing between “suspected” and “confirmed” intrauterine inflammation [2]. For clinical diagnosis, according to the ACOG Clinical Practice Update (July 2024), suspected intraamniotic infection is diagnosed when maternal temperature is ≥39.0 °C, or when temperature is 38.0–38.9 °C alongside at least one additional clinical risk factor, including fetal tachycardia >160 bpm, maternal leucocytosis >15,000/mm^3^ in the absence of corticosteroid therapy, or purulent cervical discharge [3]. It is now recognized that the syndrome encompasses three distinct etiologies: true intraamniotic infection, sterile intraamniotic inflammation in the absence of demonstrable microorganisms, and systemic maternal inflammation induced by epidural analgesia [4,5].

Despite these clinical criteria, a significant proportion of chorioamnionitis cases remain asymptomatic and are detectable only on histological examination of the placenta. Histological chorioamnionitis refers to acute placental inflammation characterized by neutrophilic infiltration across multiple sites within the organ [6]. Building on the framework established by the Perinatal Section of the Society of Pediatric Pathology [7], the Amsterdam Placental Workshop Group Consensus Statement introduced a structured staging system for maternal inflammatory responses, distinguishing three levels of escalating severity [8]. Stage I, termed acute subchorionitis or chorionitis, marks the earliest phase, with inflammatory changes confined to the chorion. As the process advances, stage II, or acute chorioamnionitis, develops when inflammation extends beyond the chorionic plate into the fibrous chorion and/or the amnion. In its most severe form, stage III, or necrotising chorioamnionitis, the inflammatory response produces frank cellular injury and tissue necrosis affecting both the amnion and chorion.

Fetal Inflammatory Response Syndrome (FIRS) represents the fetal counterpart of the systemic inflammatory response syndrome, first described in adults. The primary diagnostic criterion is an elevation of blood IL-6 > 11 pg/mL in fetal blood, with characteristic multisystem involvement. FIRS typically arises in the context of chorioamnionitis, and almost 50% of pregnancies complicated by histological and/or clinical chorioamnionitis result in premature delivery [4,9]. A study of 1116 newborns demonstrated that FIRS is associated with a higher rate of neonatal complications (including early neonatal sepsis, bronchopulmonary dysplasia, intraventricular haemorrhage, periventricular leukomalacia, acute respiratory distress syndrome, and neonatal death) compared to those without FIRS [10].

Neonatal complications associated with chorioamnionitis, and FIRS are classified as either short-term or long-term. Short-term complications include early neonatal sepsis, necrotising enterocolitis, retinopathy of prematurity, immunodeficiency, bronchopulmonary dysplasia, acute respiratory distress syndrome, white matter lesions, and intraventricular haemorrhage. Long-term complications include asthma, chronic obstructive respiratory diseases, cerebral palsy, developmental delay, hearing loss, and autism spectrum or schizophrenia disorders [4,11].

This study aimed to determine the prevalence of chorioamnionitis in a cohort of extremely preterm infants and to assess its association with neonatal morbidity and mortality in a tertiary centre.

## 2. Materials and Methods

### 2.1. Characteristics of the Study

This observational, retrospective study was conducted over three years (2019–2021) in the Neonatology I Department of Cluj County Emergency Clinical Hospital. The study population consisted of preterm infants born at 23 to 28 weeks of gestational age who were admitted to the neonatal intensive care unit. Of the 109 subjects initially enrolled, 29 were excluded due to missing placental histopathological examination results, yielding a final sample of 80 subjects (40 male and 40 female). Participants were then divided into two subgroups: those with exposure to chorioamnionitis (65/80) and those without (15/80). All data were extracted from patients’ medical records and handled in strict accordance with confidentiality and anonymity regulations.

### 2.2. Clinical Data

In the current study we analysed the association of chorioamnionitis exposure with in-hospital neonatal morbidities and mortality. The neonatal morbidities analysed were respiratory distress (RDS) and Silverman score for severity of the disease, intraventricular haemorrhage (IVH), gastrointestinal haemorrhage, pulmonary haemorrhage, pneumothorax, early onset sepsis (EOS), necrotising enterocolitis (NEC), bronchopulmonary dysplasia (BPD), and retinopathy of prematurity (ROP). All in-hospital complication diagnoses were extracted from the medical records of the enrolled patients.

Furthermore, the study examined the association between maternal conditions and neonatal morbidities. Maternal and pregnancy variables studied were cervical cerclage, placenta previa, oligohydramnios, pre-eclampsia, pregnancy-induced hypertension, and urinary tract infection prior to delivery. For C-reactive protein (CRP) determination, we used the immunoturbidimetry method with a clinical chemistry analyzer (Beckman Coulter, AU 680, Brea, CA, USA).

### 2.3. Statistical Analysis

Continuous variables were summarized as mean ± standard deviation or median (interquartile range), as appropriate. Comparisons between groups were performed using Student’s *t*-test or Mann–Whitney U test for continuous variables and between categorical variables were performed using Pearson’s χ^2^ test or Fisher’s exact test when expected cell counts were <5. Associations involving ordinal variables, including histological chorioamnionitis stage, Apgar scores, and Silverman scores, were evaluated using Spearman rank correlation coefficients. Logistic regression analyses were used to evaluate associations between histological chorioamnionitis and binary neonatal outcomes. Adjusted models included gestational age, birth weight, sex, antenatal corticosteroid exposure, mode of delivery, and maternal hypertensive disorders. Adjusted odds ratios (aORs) with 95% confidence intervals (95% CIs) were reported. Statistical significance was defined as *p* < 0.05.

For rank correlation analyses, histological chorioamnionitis severity was treated as an ordinal variable with stage 0 taken as absent, and stages I, II, III as present. Throughout this paper, the term “histological CA stage I–II–III” is used to differentiate degrees of severity, and “with/without CA” to denote the presence or absence of histological chorioamnionitis. Given the exploratory nature of this study and the limited sample size, formal correction for multiple testing was not applied. The analyses were intended to generate hypotheses regarding potential associations between histological chorioamnionitis and neonatal outcomes. Therefore, *p*-values should be interpreted cautiously, particularly for findings close to the conventional threshold for statistical significance. Statistical analyses were performed using IBM SPSS Statistics for Windows, Version 25.0 (IBM Corp., Armonk, NY, USA) and R software, version 4.4.1.

## 3. Results

### 3.1. Study Population and Clinical vs. Histological Chorioamnionitis Prevalence

Clinical chorioamnionitis was identified in 12 of 80 (15.0%) preterm infants enrolled. In contrast, histological examination of the placenta revealed a significantly higher prevalence, with 81.3% (65 out of 80) of newborns showing signs of chorioamnionitis. Out of the 12 cases of clinical chorioamnionitis, 11 were confirmed by histopathological examination of the placenta retrospectively.

Table 1 presents an overview of the baseline demographic and perinatal characteristics of the study group. Most of the demographic and perinatal variables were comparable between groups, with the exception of PROM, which was significantly more frequent in the CA-exposed group (46.2% vs. 13.3%, *p* = 0.019), and PROM duration (*p* = 0.02). We found no significant differences in demographic variables between the chorioamnionitis-exposed and non-exposed groups (*p* > 0.05).

### 3.2. Maternal and Pregnancy Characteristics by Chorioamnionitis Exposure

Cervical cerclage, placenta praevia, and oligohydramnios were observed exclusively in the CA-exposed group (17.0%, 2.1%, and 4.3% respectively), with none recorded in the non-exposed group, though these differences should be interpreted cautiously given the small sample size. Pre-eclampsia and pregnancy-induced hypertension were more frequent in the exposed group (6.7% vs. 7.7%; 6.7% vs. 9.2%), though differences were not statistically relevant (*p* = 1 for both pre-eclampsia and pregnancy-induces hypertension). The urinary tract infection was more frequent in the non-exposed group, (20% vs. 6.2%), but statistically not significant (*p* = 0.12).

### 3.3. Chorioamnionitis Stage and Gestational Age

**Figure 1:**
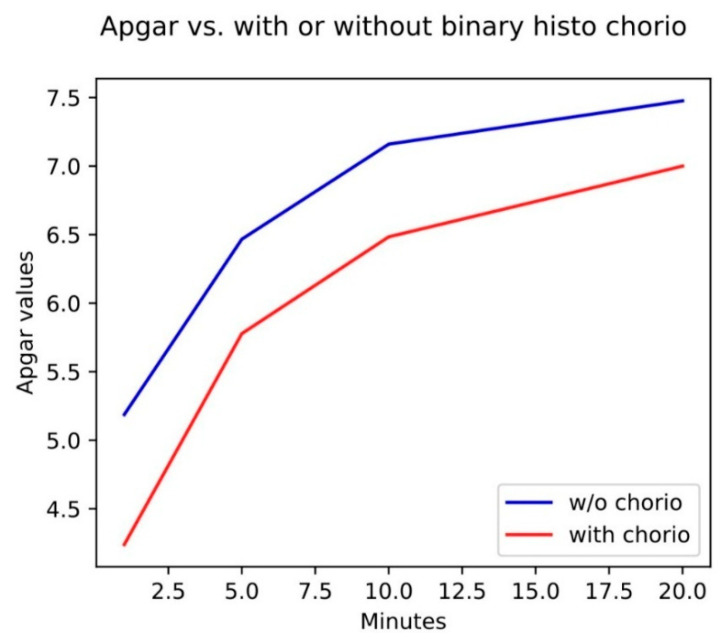
Apgar score evolution according to chorioamnionitis exposure.

Although higher histological chorioamnionitis stages tended to occur among infants with lower gestational age, this association was weak and did not reach statistical significance (Spearman ρ = −0.15, *p* = 0.195). Descriptively, cases at the most extreme prematurity (23–24 weeks) were predominantly found in higher CA stages, although the relationship was not uniform across all stages and the overall association did not reach statistical significance (Figure 1).

### 3.4. Neonatal Transition and Apgar Scores

Table 2 shows the associations between histological chorioamnionitis stage and Apgar scores. Negative coefficients indicate lower values of the analysed variable with increasing chorioamnionitis severity, whereas positive coefficients indicate higher values with increasing severity.

The neonatal transition quantified by the Apgar score over the first twenty minutes after birth highlights a different dynamic in newborns exposed to chorioamnionitis compared to those born without the exposure to the inflammatory environment. From the first minute of life, a significant difference is observed: the median Apgar score at 1 min was lower among infants exposed to histological chorioamnionitis compared with non-exposed infants [5.0 (IQR 3.0–6.0) vs. 6.0 (IQR 4.5–7.0)].

**Figure 2:**
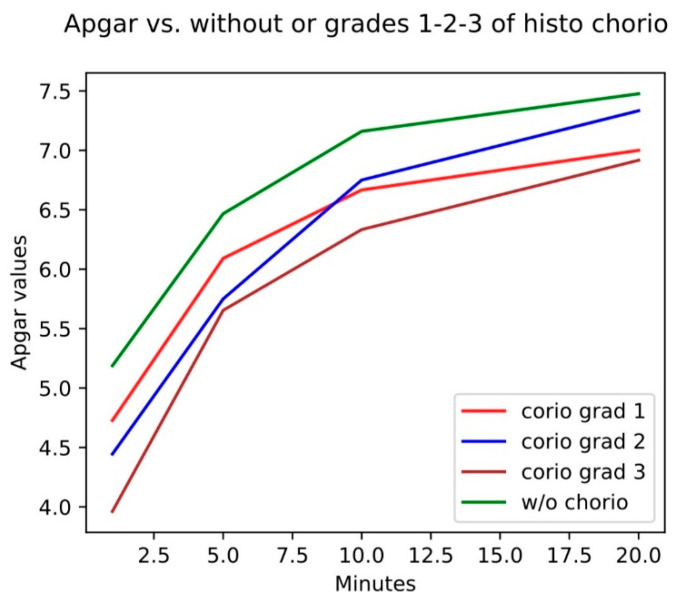
Apgar score and severity of histological chorioamnionitis (stages I, II or III).

This disparity persisted over the next few minutes, with the curve of newborns exposed to CA remaining consistently below that of unexposed newborns. Nevertheless, we note a narrowing of this gap around twenty minutes, though CA-exposed infants remained below their non-exposed counterparts (Figure 2).

### 3.5. Mortality and Morbidities of Study Group

Among the 80 infants included in the study, 25 (31.3%) died during hospitalization. Mortality was numerically higher among infants without histological chorioamnionitis than among those exposed to histological chorioamnionitis (46.7% vs. 27.7%); however, this difference did not reach statistical significance (*p* = 0.216, Fisher’s exact test).

Severe respiratory distress syndrome represented the most frequent neonatal morbidity, followed by intraventricular haemorrhage, early-onset sepsis, pulmonary haemorrhage, and gastrointestinal haemorrhage. Neonatal outcomes according to histological chorioamnionitis exposure are presented in Table 3.

Infants exposed to histological chorioamnionitis tended to experience higher rates of several adverse neonatal outcomes, including early-onset sepsis (46.2% vs. 26.7%), intraventricular haemorrhage (73.8% vs. 60.0%), bronchopulmonary dysplasia (15.9% vs. 6.7%), and retinopathy of prematurity (11.3% vs. 0.0%). The incidence of necrotising enterocolitis was comparable between groups (14.1% vs. 13.3%). Pneumothorax occurred more frequently among infants without histological chorioamnionitis (14.3% vs. 1.5%). However, most of these differences did not reach statistical significance in unadjusted analyses.

Respiratory morbidity was particularly prominent among infants exposed to histological chorioamnionitis. Severe respiratory distress syndrome occurred significantly more frequently in the exposed group than in the non-exposed group (90.8% vs. 60.0%, *p* = 0.008).

To further evaluate whether histological chorioamnionitis independently predicted neonatal morbidity and mortality, multivariable logistic regression analyses were subsequently performed (Table 4).

Multivariable logistic regression analyses were performed to evaluate whether histological chorioamnionitis independently predicted adverse neonatal outcomes after adjustment for gestational age, birth weight, sex, antenatal corticosteroid exposure, mode of delivery, and maternal hypertensive disorders. Several associations observed in unadjusted analyses were attenuated after adjustment for these confounding factors. However, histological chorioamnionitis remained independently associated with severe respiratory distress syndrome (aOR 25.84, 95% CI 2.49–268.44, *p* = 0.006). No statistically significant independent associations were identified for mortality, early-onset sepsis, intraventricular haemorrhage, bronchopulmonary dysplasia, necrotising enterocolitis, pulmonary haemorrhage, gastrointestinal haemorrhage, or pneumothorax. Consistent with the systemic inflammatory response observed across these morbidities, C-reactive protein (CRP) measured on the first day of life showed a significant positive correlation with histological CA stage (*p* < 0.05), supporting its potential utility as an early indicator of chorioamnionitis exposure.

## 4. Discussion

This study demonstrated that the risk of neonatal complications is higher in preterm infants exposed to histological chorioamnionitis than in non-exposed ones. Notably, the mortality of patients exposed to histological chorioamnionitis was not significantly associated with CA exposure. Despite comparable gestational ages between the two groups (Table 1), the mortality rate was lower in the CA-exposed group (27.7%) than in the cohort overall (31.3%), a counterintuitive finding for which the most likely explanation is limited statistical power given the small sample size. The incidence and severity of acute complications such as RDS or IVH were higher in CA-exposed infants than in non-exposed ones.

Olguin et al. reported an early death incidence of 3.5% and stillbirth rate of 14%, though their cohort had a significantly higher gestational age (30.2 ± 5.4 weeks and 32.5 ± 5.1 weeks) than our study population of ≤28 weeks, limiting direct comparability [12]. Yu et al., in a Chinese cohort of pregnancies before 34 weeks, reported a clinically diagnosed chorioamnionitis incidence of 17.8% and a neonatal mortality rate of 7.4%, with major neonatal complications occurring in 40% of cases [13]. The higher mortality rates in our cohort likely reflect the more extreme prematurity of our study population rather than a specific effect of chorioamnionitis exposure. In unadjusted analyses, histological chorioamnionitis was associated with several neonatal complications including respiratory distress, early onset sepsis, bronchopulmonary dysplasia, and retinopathy of prematurity, though only severe RDS remained independently significant after multivariable adjustment.

The disparity between clinical and histological chorioamnionitis (15% vs. 81.3%) prevalence in our cohort suggests that clinical diagnostic criteria, such as the presence of fever, tachycardia, and purulent cervical discharge, have good specificity, but poor sensitivity. This likely reflects, on the one hand, the large proportion of cases that remain asymptomatic, despite harbouring histological evidence of chorioamnionitis, a subclinical course that most likely reflects detection at an early, pre-symptomatic stage of the underlying process; and the limited ability to make a histological diagnosis antenatally, on the other. These results highlight the need for improved chorioamnionitis diagnostic tools, including highly sensitive maternal biomarkers, or the combined use of clinical, biological, and ultrasound criteria, which would extend detection to subclinical cases that are missed or underdiagnosed. Notably, histological chorioamnionitis is not exclusively of infectious origin: in the absence of any microbiological evidence, the condition can arise through sterile intra-amniotic inflammation, driven by danger signals released during cellular stress, injury, or death [14,15,16,17].

Placental histopathological examination carries significant diagnostic value in pregnancies complicated by premature rupture of membranes (PROM), prolonged PROM, preterm delivery, maternal fever, maternal leucocytosis, fetal tachycardia, or purulent cervical discharge, and should be considered standard practice in this clinical context.

Our study identified significant negative correlations with Apgar scores at 1 min (ρ = −0.25, *p* = 0.029), 10 min (ρ = −0.36, *p* = 0.0069), and 20 min (ρ = −0.35, *p* = 0.0246) consistent with the high prevalence of histological chorioamnionitis (81.3%) observed in this extremely preterm cohort.

Although higher histological CA stages tended to occur at lower gestational ages, this association did not reach statistical significance (Spearman ρ = −0.15, *p* = 0.195). The high prevalence of histological chorioamnionitis in this extremely preterm cohort (81.3%) is itself consistent with the well-established link between intrauterine inflammation and preterm birth, even in the absence of a statistically significant stage-GA correlation. Fahmi et al. described a significant correlation between high-grade histologic chorioamnionitis and spontaneous preterm birth in a Swedish cohort [18]; while a similar trend was observed in our data, it did not reach statistical significance, possibly reflecting the limited statistical power of the present work. Overall, intrauterine inflammatory exposure is a widely described risk factor for preterm birth and subsequent perinatal complications [19]; in a previous study from our group, a significant correlation was found between early inflammatory markers and the incidence of NEC [20].

CA-exposed newborns were at increased risk of most neonatal complications examined. While unadjusted analyses showed associations between histological chorioamnionitis and early onset sepsis, respiratory distress syndrome, bronchopulmonary dysplasia, and retinopathy of prematurity, only severe RDS remained independently significant after multivariate analysis. EOS occurred more frequently in CA-exposed neonates (46.2% vs. 26.7%). The meta-analysis by Beck et al. found that both histological and clinical chorioamnionitis were associated with significantly increased odds of confirmed and early-onset neonatal sepsis, and with higher odds of late-onset sepsis in preterm neonates [14], supporting our findings. Our study examined only early onset sepsis; the association with late-onset sepsis warrants consideration in future studies of this population. IVH was also more frequent in the CA-exposed group (73.8% vs. 60.0%), as was retinopathy of prematurity (11.3% vs. 0.0%). The incidence of NEC was comparable between the groups (14.1% vs. 13.3%), though none of these differences reached statistical significance in the analysis; these are findings that merit investigation in larger cohorts.

Severe RDS was particularly prominent among infants exposed to histological chorioamnionitis (90.8% vs. 60.0%, *p* = 0.008). Exposure of extremely preterm neonates to intrauterine maternal inflammation may affect lung development and neonatal outcomes and may contribute to the development of BPD.

BPD was substantially more common in CA-exposed infants in our cohort (15.9% vs. 6.7%) but did not reach statistical significance. Villamor-Martinez et al., in their meta-analysis of 244,000 infants concluded that chorioamnionitis increased the risk of BPD [21], though reports from the Canadian Neonatal Network and Laughon et al. found no such association [22]. A plausible reconciliation is the two-hit model: chorioamnionitis provides the first inflammatory hit, with subsequent postnatal mechanical ventilation and oxygen exposure constituting the second hit. Evidence supports this, with BPD risk decreased overall in ventilated CA-exposed infants but increased in those infants ventilated for more than seven days or with postnatal sepsis [23,24,25].

These findings collectively highlight the importance of increased surveillance of extremely preterm infants, regardless of whether chorioamnionitis was clinically suspected, given the poor sensitivity of clinical diagnostic criteria and the significant morbidity burden associated with histological exposure. Early detection and appropriate management of chorioamnionitis in pregnant women may reduce the risk of preterm birth and its associated complications.

The significant positive correlation observed between day-of-life C-reactive protein (CRP) and histological CA stage (*p* < 0.05) reflects the systemic inflammatory response associated with chorioamnionitis exposure in the neonatal period. CRP and more specific biomarkers such as interleukins support the concept of chorioamnionitis as a first inflammatory hit that contributes to preterm birth and subsequent neonatal morbidity [11,26]. At the maternal level, both procalcitonin (PCT) and CRP are appropriate biomarkers in predicting subclinical intrauterine infection in women with PROM before 34 weeks of gestation, with PCT particularly applicable between 28 and 33 + 6 weeks [27]. A retrospective Swedish cohort study of 500 term singleton deliveries found that elevated inflammatory markers were associated with both neonatal infection and asphyxia-related complications, supporting the inclusion of maternal CRP into the clinical management of chorioamnionitis [14].

Our results are broadly consistent with those reported in a study conducted at a provincial perinatal centre in China, in 2014–2015 [19]. Although their sample size was substantially larger, with 14,166 cases compared to 80 in our study, the findings converge on several key points. Both studies demonstrated that histological chorioamnionitis was significantly associated with various neonatal complications, and that histological assessment had greater diagnostic value in predicting complications than clinical assessment alone. Both also support the importance of routine histological examination of the placenta. However, unlike our study, the Chinese study observed a significantly higher neonatal mortality rate in histologically CA-exposed newborns compared to non-exposed ones, a discrepancy that may reflect differences in sample size, gestational age distribution, or local clinical management practices, and that warrants further investigation.

An interesting finding of the present study was the numerically lower mortality observed among infants exposed to histological chorioamnionitis, despite the increased burden of respiratory morbidity. Although this difference did not reach statistical significance and histological chorioamnionitis was not independently associated with mortality after multivariable adjustment, several explanations may be considered. First, intrauterine inflammatory exposure has been associated with accelerated fetal lung maturation through cytokine-mediated stimulation of surfactant production and pulmonary development, potentially improving postnatal adaptation despite increasing respiratory morbidity. In addition, the observed mortality pattern may reflect residual confounding and random variation related to the relatively small sample size rather than a true protective effect of histological chorioamnionitis. Similar observations have been reported in previous studies, supporting the concept that intrauterine inflammation may differentially influence neonatal morbidity and mortality depending on gestational age, inflammatory severity, and associated maternal or placental conditions [25,26].

This study has several limitations. First, the study population was restricted to infants born at 23–28 weeks of gestation, and no comparison was made with less preterm or term infants. This limits the ability to disentangle complications attributable to extreme prematurity from those specifically related to chorioamnionitis exposure and should be considered when interpreting the findings.

Second, although multivariable analyses were performed adjusting for gestational age, birth weight, sex, antenatal corticosteroid exposure, mode of delivery, and maternal hypertensive disorders, several clinically relevant variables were unavailable in the study database, including maternal antibiotic treatment for chorioamnionitis, funisitis assessment, and fetal inflammatory response syndrome data. Several associations observed in unadjusted analyses were attenuated after adjustment, underscoring the importance of baseline perinatal characteristics in determining outcome. However, the association between histological chorioamnionitis and severe respiratory distress syndrome persisted after multivariable adjustment, and the wide confidence interval observed (aOR 25.84, 95% CI 2.49–268.44) reflects the limited sample size and should be interpreted with caution.

Third, this study should be interpreted as exploratory in nature. The evaluation of multiple maternal, inflammatory, and neonatal variables increases the risk of chance findings, particularly among associations with modest effect sizes and borderline *p*-values. Consequently, these results should be considered hypothesis-generating and require validation in larger, adequately powered prospective studies before definitive conclusions can be drawn.

Fourth, data on the temporality of events were not assessed, including the onset of early versus late neonatal sepsis, and the precise timing of death. Early neonatal sepsis is more directly related to chorioamnionitis, while late sepsis is more commonly a consequence of prematurity itself. Similarly, early death is more likely to reflect the direct impact of chorioamnionitis, whereas death at an older gestational age may be more attributable to the consequences of premature birth. The absence of this temporal data limits the interpretation of the mortality findings.

Finally, the small overall sample size limits statistical power and the generalisability of several findings. This is particularly relevant for the pre-eclampsia, and mortality associations, where the number of affected cases within subgroups is very small; these findings should be interpreted accordingly.

## 5. Conclusions

In unadjusted analyses, histological chorioamnionitis was associated with higher rates of early-onset sepsis, severe respiratory distress syndrome, bronchopulmonary dysplasia, and retinopathy of prematurity. After multivariable adjustment, histological chorioamnionitis remained independently associated with severe respiratory distress syndrome (aOR 25.84, 95% CI 2.49–268.44, *p* = 0.006), suggesting that intrauterine inflammation contributes independently to early respiratory compromise in extremely preterm infants. No significant association was found between the presence or severity of histological chorioamnionitis and neonatal mortality. The clinical diagnostic criteria for chorioamnionitis demonstrated good specificity but poor sensitivity, highlighting the need for improved diagnostic tools.

## Figures and Tables

**Table 1:** Baseline demographic and perinatal characteristics according to histological chorioamnionitis exposure.

Variable	Without Histological CA (n = 15)	With Histological CA (n = 65)	*p*-Value
Gestational age, (weeks) †	26.0 (25.0–26.0)	26.0 (25.0–27.0)	0.313
Birth weight(g) †	780 (695–845)	740 (690–890)	0.946
Length (cm) *	34.0 ± 2.8	34.2 ± 3.7	0.804
Head circumference (cm) †	24.0 (23.0–25.0)	24.0 (22.0–25.0)	0.783
Ponderal index †	2.0 (1.8–2.4)	2.1 (1.9–2.3)	0.707
Male sex, n (%)	10 (66.7%)	30 (46.2%)	0.152
Antenatal corticosteroid exposure, n (%)	10 (66.7%)	56 (86.2%)	0.124
Complete antenatal corticosteroid course, n (%)	9 (60.0%)	42 (64.6%)	0.737
Caesarean delivery, n (%)	9 (60.0%)	32 (49.2%)	0.452
Preeclampsia, n (%)	1 (6.7%)	5 (7.7%)	1
Pregnancy-induced hypertension, n (%)	1 (6.7%)	6 (9.2%)	1
Maternal hypertensive disorder, n (%)	1 (6.7%)	11 (16.9%)	0.447
Urinary tract infection (UTI), n (%)	3 (20.0%)	4 (6.2%)	0.12
PROM, n (%)	2 (13.3%)	30 (46.2%)	0.019
PROM ≥ 18 h, n (%)	1 (6.7%)	21 (32.3%)	0.056
PROM duration, hours †	0.0 (0.0–0.0)	0.0 (0.0–31.0)	0.02
Surfactant administration, n (%)	15 (100.0%)	65 (100.0%)	—
Mechanical ventilation, hours †	156.0 (24.0–258.0)	96.0 (44.0–192.0)	0.523

* Values are presented as mean ± standard deviation. † Values are presented as median (interquartile range, IQR). PROM = premature rupture of membranes. *p*-values: Student’s *t*-test, Mann–Whitney U test, χ^2^ or Fisher’s exact test, as appropriate.

**Table 2:** Association between histological chorioamnionitis stage and Apgar scores.

Correlated Variables	Spearman ρ	*p*-Value
Histological CA stage vs. Apgar 1 min	−0.25	0.029
Histological CA stage vs. Apgar 10 min	−0.36	0.0069
Histological CA stage vs. Apgar 20 min	−0.35	0.0246

Histological chorioamnionitis severity showed significant negative correlations with Apgar scores at 1 min (ρ = −0.25, *p* = 0.029), 10 min (ρ = −0.36, *p* = 0.0069), and 20 min (ρ = −0.35, *p* = 0.0246). These findings suggest impaired neonatal transition among infants exposed to more severe intrauterine inflammation, with effects persisting beyond the immediate postnatal period.

**Table 3:** Neonatal outcomes according to histological CA exposure.

Outcome	Without Histological CA (n = 15)	With Histological CA (n = 65)	*p*-Value
Mortality	7/15 (46.7%)	18/65 (27.7%)	0.216
Early onset sepsis (EOS)	4/15 (26.7%)	30/65 (46.2%)	0.169
RDS-severe	9/15 (60.0%)	59/65 (90.8%)	0.008
IVH, any grade	9/15 (60.0%)	48/65 (73.8%)	0.346
Severe IVH, grade III–IV	4/15 (26.7%)	11/65 (16.9%)	0.464
Pulmonary haemorrhage	5/14 (35.7%)	20/65 (30.8%)	0.757
Gastrointestinal haemorrhage	7/14 (50.0%)	17/65 (26.2%)	0.109
Pneumothorax	2/14 (14.3%)	1/65 (1.5%)	0.079
NEC	2/15 (13.3%)	9/64 (14.1%)	1
BPD	1/15 (6.7%)	10/63 (15.9%)	0.681
ROP	0/14 (0.0%)	7/62 (11.3%)	0.337

RDS: respiratory distress syndrome; EOS: early onset sepsis; BPD: bronchopulmonary dysplasia; NEC: necrotising enterocolitis; ROP: retinopathy of prematurity.

**Table 4:** Crude and adjusted associations between histological chorioamnionitis and neonatal outcomes.

Outcome	Crude OR (95% CI)	*p*-Value	Adjusted OR (95% CI) *	*p*-Value
Mortality	0.44 (0.14–1.38)	0.159	0.58 (0.15–2.27)	0.432
Early-onset sepsis	2.36 (0.68–8.18)	0.177	2.75 (0.73–10.36)	0.135
RDS severe	6.56 (1.73–24.82)	0.006	25.84 (2.49–268.44)	0.006
Intraventricular haemorrhage (any grade)	1.88 (0.58–6.08)	0.29	2.63 (0.64–10.77)	0.177
Severe IVH (grade III–IV)	0.56 (0.15–2.09)	0.388	0.62 (0.10–3.67)	0.595
Necrotising enterocolitis	1.06 (0.20–5.52)	0.941	NE †	—
Bronchopulmonary dysplasia	2.64 (0.31–22.41)	0.373	4.79 (0.32–70.70)	0.254
Pulmonary haemorrhage	0.80 (0.24–2.69)	0.719	1.17 (0.31–4.37)	0.812
Gastrointestinal haemorrhage	0.35 (0.11–1.16)	0.086	0.37 (0.09–1.45)	0.154
Pneumothorax	0.09 (0.01–1.12)	0.061	0.01 (0.00–3.32)	0.12
Retinopathy of prematurity	NE †	—	NE †	—

* Adjusted for gestational age, birth weight, sex, antenatal corticosteroid exposure, mode of delivery, and maternal hypertensive disorders (pregnancy-induced hypertension and/or preeclampsia). † NE = not estimable because of complete or quasi-complete separation resulting from the low number of events.

## Data Availability

All data generated or analysed during this study are included in this published article.
